# Time to BCR and cancer-specific mortality after radiotherapy or prostatectomy: implications for risk stratification and follow-up

**DOI:** 10.1007/s00345-026-06231-5

**Published:** 2026-02-05

**Authors:** Francesco Pellegrino, Ralph Grauer, Ugo Giovanni Falagario, Olof Akre, Markus Aly, Tobias Nordström, Henrik Grönberg, Lars Egevad, Ash Tewari, Matteo Ferro, Giorgio Gandaglia, Alberto Briganti, Francesco Montorsi, Anna Lantz, Peter N. Wiklund

**Affiliations:** 1 Department of Urology, ASST Santi Paolo E Carlo, Milan, Italy; 2 Department of Molecular Medicine and Surgery, Karolinska Institute, Stockholm, Sweden; 3 Department of Urology, Icahn School of Medicine at Mount Sinai, New York, NY USA; 4 Department of Urology and Organ Transplantation, University of Foggia, Foggia, Italy; 5 Departments of Medical Epidemiology and Biostatistics, Karolinska Institutet, Stockholm, Sweden; 6 Department of Clinical Sciences, Danderyd Hospital, Karolinska Institutet, Stockholm, Sweden; 7 Department of Oncology and Pathology, Karolinska Institute, Stockholm, Sweden; 8 Unit of Urology/Division of Oncology, Soldera Prostate Cancer Lab, Urological Research Institute, IRCCS San Raffaele Hospital, Milan, Italy

**Keywords:** Prostate cancer, Biochemical recurrence, PSA, Prostatectomy, Radiotherapy

## Abstract

**Purpose:**

To evaluate the association between time to biochemical recurrence (ttBCR) and cancer-specific mortality (CSM) after prostatectomy or radiotherapy.

**Methods:**

This population-based study included 3606 patients experiencing BCR after prostatectomy or radiotherapy in Stockholm, Sweden, between 2003 and 2019. We tested the association between ttBCR and CSM using Cox models adjusted for clinical and pathological characteristics and estimated conditional cancer-specific survival by ttBCR and EAU risk group.

**Results:**

Among prostatectomy (n = 2392) vs radiotherapy patients (n = 1214), 589 vs 170 (25 vs 14%), 1205 vs 387 (50 vs 32%), 598 vs 657 (25 vs 54%) were low, intermediate, and high EAU risk group, respectively. The median ttBCR was 29 (interquartile range (IQR):11, 58) and 33 months (IQR:16, 60) among prostatectomy and radiotherapy patients, respectively. At a median follow-up for survivors of 52 months after BCR, 823 patients died, including 394 of PCa (104 in the prostatectomy and 290 in the radiotherapy group). At Cox multivariable models, ttBCR was associated with CSM in prostatectomy patients (HR:0.87; p:0.012) and radiotherapy patients (HR:0.69; p < 0.001). Adjusted cancer-specific survival probability was strongly influenced by ttBCR and EAU risk group. For instance, intermediate-risk patients with BCR after prostatectomy had a 10-year survival probability ranging from 0.74 to 0.98 for a ttBCR of 0 to 10 years. Limitations included the retrospective nature of the study.

**Conclusions:**

These findings demonstrate a strong association between ttBCR and CSM. These results suggest that ttBCR should be used to risk-stratify patients experiencing BCR after either RP or RT. The high survival probability observed in low- and intermediate-risk PCa patients who experience BCR after a prolonged period post-treatment should inform decisions regarding the intensity and duration of oncological follow-up for these patients.

**Supplementary Information:**

The online version contains supplementary material available at 10.1007/s00345-026-06231-5.

## Introduction

Prostate cancer (PCa) is estimated to affect 1 in 8 men and a total of 288,300 men in the United States in 2023[1]. Biochemical recurrence (BCR) is defined as PSA ≥ 0.2 ng/mL after radical prostatectomy (RP), and PSA ≥ 2 + PSA nadir after radiation therapy (RT) [2–6]. BCR occurs in approximately 15–40% at 10–15 years of follow-up either after RP or RT [7–9]. Despite the relatively high rates of BCR, the natural history of BCR does not necessarily lead to clinical metastatic disease, and thus PCa-specific mortality is low—less than 3% at 15 years regardless of treatment modality [10]. Considering that salvage treatments such as androgen deprivation (ADT) and external beam radiation carry side effects[11, 12], it is crucial to identify patients at low risk of death who may not benefit from these therapies. The European Association of Urology (EAU) guidelines suggest stratifying patients experiencing BCR into a high-risk or low-risk group based on pathological characteristics (ISUP grade), PSA doubling time, and time to BCR (ttBCR), the latter used as a categorical variable (< or > 18 months only for stratifying patients experiencing BCR after RT). However, these risk stratification systems showed only moderate discrimination [8, 13]. Moreover, current guidelines do not propose an appropriate duration of follow-up after treatment. The systematic review that informed the most recent EAU guidelines summarized the relationship between ttBCR and distant metastasis, prostate cancer-specific mortality (CSM), and overall mortality after RP and RT in several studies[14]. However, most studies on this topic relied on small populations, they did not evaluate ttBCR after stratifying patients according to disease characteristics, and only a few of these studies utilized ttBCR as a continuous variable [15–21] making the effect size less interpretable due to the variance in ttBCR cutoffs.

To fill these voids, we aimed to evaluate how the risk of dying from prostate cancer varies according to ttBCR in a population-based cohort of patients who experienced BCR after either RT or RP, stratified according to disease characteristics.

## Methods

### Study population

This retrospective cohort study used the Stockholm PSA and Biopsy Register (STHLM-0), a population-based registry that contains data on every PSA test and prostate biopsy taken in Stockholm County since 2003 and includes 17,017 PCa patients. This study was approved by the local ethics board in Stockholm, Sweden, and specific informed consent was not required given the nature of deidentified data from a population-based registry. The study was reported following the Strengthening the Reporting of Observational Studies in Epidemiology (STROBE) reporting guideline. All patients who underwent RP or RT with curative intent (≤ cT3, cM0) and experienced BCR (defined as a PSA ≥ 0.2 ng/mL in two consecutive measurements after RP, and as an increase ≥ 2 ng/mL above the post-radiation PSA nadir after RT[3, 4]) were included; thus, resulting in a final population of 3606 patients. The decision to perform RP or RT and any additional therapies was left to the clinical judgment of the treating physician after discussion with each patient. PSA measurements were performed in three centralized laboratories and were linked to the National Prostate Cancer Register (NPCR) of Sweden, the Prescribed Drug Register, the inpatient and outpatient registries, and the Swedish Cause of Death Register [22]. Methods for assessment of cause of death in Swedish studies have been previously described [23]. Follow-up for all patients was until death, emigration, or end of the study period. The study end date was set to the last available update of the Swedish Death Register (March 2021).

### Statistical analysis

The primary outcome of the study was CSM. Statistical analyses consisted of several steps. First, we described patient characteristics stratified by initial treatment. Since the aim of the study was not to compare treatment efficacy, all analyses were performed separately for patients who underwent RT and those who underwent RP. We tested if ttBCR was associated with CSM using Cox multivariable models. Cox models were adjusted for potential confounders such as PSA at BCR (BCR-PSA), clinical T stage (cT1 vs cT2 vs cT3), clinical ISUP grade group (1 vs 2 vs 3 vs ≥ 4), PSA at diagnosis, year of treatment, age at BCR, and adjuvant ADT (yes or no). We evaluated whether or not the association between ttBCR and CSM was linear. We used restricted cubic splines for ttBCR with four knots placed at the 5th, 35th, 65th, and 95th percentiles. Since there was evidence of a nonlinear relationship between ttBCR and CSM (p < 0.05), the nonlinear terms were retained for the following steps [24]. Then we plotted the adjusted cancer-specific survival probability for patients grouped by treatment modality and EAU risk group [4]. To compare the CSM by ttBCR with the overall mortality rate, we plotted the 10-year CSM risk by ttBCR predicted with our model with the 10-year overall mortality risk of Swedish men by age using data from Statistics Sweden (http://www.scb.se). As supplementary analyses, using a subset of patients that underwent RP and complete pathological information (n = 1798 patients), an additional multivariable Cox model was constructed to assess the association between ttBCR and CSM adjusting for BCR-PSA, year of treatment, adjuvant ADT (yes or no), and pathological characteristics such as pathological ISUP grade group (< 3 vs ≥ 3), pathological T stage (pT < 3 or pT ≥ 3), surgical margins status (positive or negative), pathological N stage (pN1 or pN0-X), and age at BCR. We plotted the adjusted cancer-specific survival probability for patients grouped by CAPRA-S score [25]. Finally, we tested the association between ttBCR and CSM among patients who underwent RT after excluding patients who did not receive ADT. All tests were two-sided with a significance level of 0.05. Statistical analyses were performed using R v4.0.2 statistical software (R Foundation for Statical Computing, Vienna, Austria).

## Results

### Patient characteristics

A total of 3606 patients underwent treatment for localized PCa with either RP (n = 2392) or RT (n = 1214). Patient and tumor characteristics stratified by treatment type are summarized in Table 1. Patients who underwent RT had worse clinical characteristics. Specifically, according to the EAU criteria, 589 vs 170 (25 vs 14%), 1205 vs 387 (50 vs 32%), 598 vs 657 (25 vs 54%) patients fell in the low-, intermediate-, and high-risk groups among patients treated with RP vs RT, respectively. The median ttBCR was 29 months (interquartile range (IQR): 11, 58) among RP patients and 33 months (IQR: 16, 60) among RT patients. The median BCR-PSA was 0.30 ng/ml (0.24, 0.52) and 4.60 ng/ml (2.90, 10.00) among RP and RT patients, respectively.

**Table 1:** Characteristics of patients who experienced biochemical recurrence (BCR) after primary treatment

Characteristic	Radical prostatectomy, N = 2392^*1*^	Radiotherapy, N = 1214^*1*^
Age at BCR, years	68 (63, 72)	73 (68, 78)
PSA at diagnosis, ng/ml	7.70 (5.20, 12.00)	12.00 (7.40, 22.95)
Clinical T stage		
1	1,412 (59%)	451 (37%)
2	846 (35%)	434 (36%)
3	134 (5.6%)	329 (27%)
ISUP grade group at biopsy		
1	779 (33%)	289 (24%)
2	802 (34%)	293 (24%)
3	449 (19%)	265 (22%)
4	213 (8.9%)	179 (15%)
5	149 (6.2%)	188 (15%)
EAU risk category		
Low risk	589 (25%)	170 (14%)
Intermediate risk	1,205 (50%)	387 (32%)
High risk	598 (25%)	657 (54%)
Year of treatment (Medin, range)	2012 (2003, 2020)	2010 (2003, 2019)
Adjuvant androgen deprivation therapy	122 (5.1%)	969 (80%)
Time to BCR, months	29 (11, 58)	33 (16, 60)
PSA at BCR, ng/ml	0.30 (0.24, 0.52)	4.60 (2.90, 10.00)
^*1*^Median (IQR); n (%); Median (Range)		

### Time-to-event analyses, RP group

Among 2392 patients who experienced BCR after RP, at a median follow-up for survivors of 53 months after BCR, 303 patients died, including 104 of PCa (sFigure 1a). In a multivariable Cox model, both ttBCR (Hazard ratio [HR] per 1-year increment: 0.87, 95% confidence intervals: 0.78, 0.97; p < 0.02, Table 2) and BCR-PSA (HR per 5 ng/ml increment: 1.04, 95% CI: 1.03, 1.04, p < 0.001 sTable 1) were associated with CSM.

**Table 2:** Point estimated and confidence intervals describing the association between time to biochemical recurrence (BCR, in years) and cancer-specific mortality in patients who experienced BCR after primary treatment derived from multivariable Cox models built in different cohorts

	Hazard ratio of time to BCR (per 1-mo increment)	95% confidence interval of time to BCR	p-value
Models based on biopsy results and clinical characteristics			
Patients experiencing BCR after RP^1^	0.87	0.78, 0.97	0.012
Patients experiencing BCR after RT^1^	0.69	0.64, 0.74	< 0.001
Model based on pathological examination results and clinical characteristics for patients experiencing BCR after RP^2^	0.68	0.58, 0.80	< 0.001

Follow-up time stated at BCR

*BCR* Biochemical recurrence, *RP* Radical prostatectomy, *RT* Radiotherapy

^1^Cox model adjusted for PSA at BCR, year of treatment, adjuvant androgen deprivation therapy (yes vs no), clinical T stage (cT1 vs cT2 vs cT3), clinical ISUP grade group (1 vs 2 vs 3 vs ≥ 4), PSA at diagnosis, and age at BCR

^2^Cox model adjusted for PSA at BCR, year of treatment, adjuvant androgen deprivation therapy (yes vs no), and pathological characteristics such as pathological ISUP grade group (< 3 vs ≥ 3), pathological T stage (pT < 3 vs pT ≥ 3), surgical margins status (positive vs negative), pathological N stage (pN1 vs pN0-X), and age at BCR

**Fig. 1:**
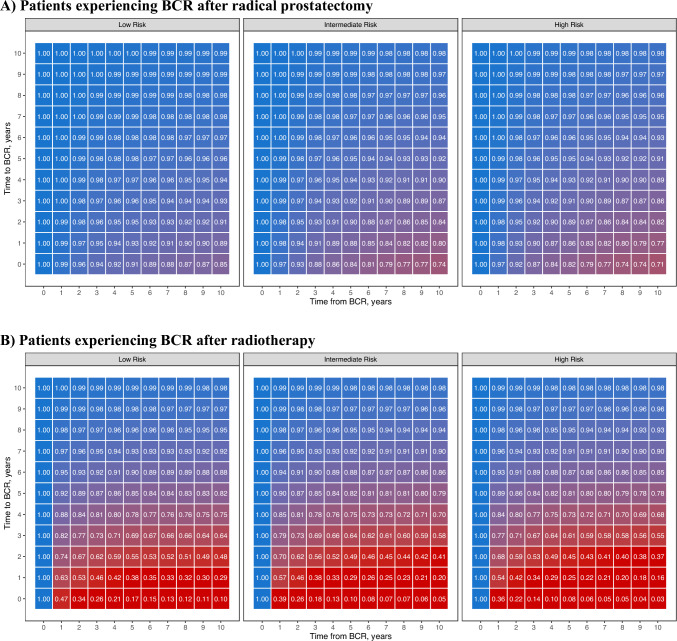
Predicted cancer-specific survival probability for patients who experienced biochemical recurrence (BCR) after primary treatment by time to BCR. Patients are grouped according to primary treatment and EAU risk group. The multivariable COX models used to calculate survival probabilities accounted for the non-linear relationship between time to BCR and cancer-specific mortality and were built with time to BCR, PSA at BCR, EAU risk group, year of primary treatment, adjuvant androgen deprivation therapy, and age at BCR

Figure 1a shows the predicted cancer-specific survival probability by ttBCR for patients grouped by EAU risk group. Notably, the survival probability at 10 years was strongly influenced by both the ttBCR and risk group. For instance, patients with intermediate-risk cancer who underwent RP and experienced BCR had a 10-year survival probability ranging from 0.74 to 0.98 for a ttBCR ranging from 0 to 10 years.

Then, we compared the 10-year CSM by ttBCR with the 10-year any-cause mortality risk according to age. The results are shown in sFigure 2a. For instance, we can observe that the risk of dying from PCa becomes lower than the risk of dying from any cause if the BCR occurs > 4 years after RP in 60–64 years old patients with intermediate-risk cancer undergoing RP.

Finally, we tested whether ttBCR was associated with CSM in patients who experienced BCR after RP using pathological information. Overall, 1798 patients treated with RP had complete pathological information and were used for this supplementary analysis (sTable 2). Among these, at a median follow-up for survivors of 49 months from BCR, 181 patients died, including 62 for PCa. Herein, ttBCR and BCR-PSA were associated with CSM at multivariable Cox analyses (ttBCR: HR per 1-year increment: 0.68; 95%CI: 0.58, 0.80; p < 0.001; BCR-PSA HR per 5 ng/ml increment: 1.03; 95% CI: 1.02, 1.04; p < 0.001, Table 2**, **sTable 3). sFigure 3 shows the predicted cancer-specific survival probability by ttBCR for patients grouped by CAPRA-S score.

### Time-to-event analyses, RT group

Among 1214 patients who experienced BCR after RT, at a median follow-up for survivors of 48 months after BCR, 520 patients died, including 290 of PCa (sFigure 1b). At multivariable Cox analyses, both ttBCR (HR per 1-year increment: 0.69, 95% CI: 0.64, 0.74, p < 0.001 Table 2) and BCR-PSA (HR per 5 ng/ml increment: 1.01, 95% CI: 1.01, 1.01, p < 0.001 sTable 1) were associated with CSM. These results were confirmed among patients who underwent RT even after excluding those who did not receive ADT (HR per 1-year increment: 0.69; 95% CI: 0.64, 0.74; p < 0.001, sTable 4). Figure 1b shows that the 10-year survival probability was strongly influenced by both ttBCR and risk group among patients who experienced BCR after RT. For instance, patients with intermediate-risk cancer who experienced BCR after RT had a 10-year survival probability ranging from 0.05 to 0.98 for a ttBCR ranging from 0 to 10 years.

Comparing the 10-year CSM by ttBCR with the 10-year any-cause mortality risk according to age (sFigure 2b), we can observe that the risk of dying from PCa becomes lower than the risk of dying from any cause if the BCR occurs > 4 years after RP in 70–74 years old patients with intermediate-risk cancer undergoing RT.

## Discussion

In this retrospective cohort study evaluating 3606 patients who experienced BCR after primary treatment for PCa with curative intent, we assessed the association between the ttBCR and CSM. Specifically, our results demonstrate that the time to recurrence significantly impacts the risk of CSM with patients experiencing BCR after a long follow-up having a very low risk of CSM. Our results emphasize that the ttBCR should be included in future prediction tools for stratifying patients experiencing BCR after treatment with curative intent. Moreover, these results can assist physicians in counseling and follow-up management for patients undergoing primary treatment for PCa.

Given that BCR is relatively common and that it does not always lead to death (CSM is less than 3% at 15 years regardless of treatment modality [10]), there is great interest in risk stratification of these patients to avoid overtreatment. The EAU guidelines have proposed a low/high risk categories for patients after undergoing RP/RT [4]. Patients experiencing BCR post-RP are considered at low risk if PSADT > 1 year and ISUP grade < 4, while high-risk patients need either PSADT < 1 year or ISUP grade 4–5. After RT, patients with BCR are considered at low risk if the interval between RT and BCR > 18 months and biopsy ISUP grade < 4 whereas high-risk patients require BCR interval < 18 months or biopsy ISUP grade 4–5. These risk groups were created based on a systematic review that found that those are the main factors that decrease survival. However, currently available models for stratifying BCR patients showed moderate discrimination (c-index ~ 0.6) pointing out the need to identify additional predictors [8–13]. In this study, we provided evidence of the association between ttBCR and CSM, suggesting that this characteristic may be used for better stratifying BCR patients both after RT (as already recommended by the guidelines) and RP. The key difference is that we used ttBCR as a continuous variable, as opposed to a cutoff value as it is used in the EAU risk classification.

Some previous studies investigated the association between ttBCR and CSM and reported conflicting results, especially regarding patients who experienced BCR after RP [14, 16, 19, 21, 26–29]. Compared to previous articles, we relied on a large population-based register that contains data on every PSA test taken in Stockholm County since 2003. Moreover, our post-treatment follow-up was notably longer than that of many other studies, with a median time from treatment to BCR of 29 months and a median follow-up after BCR for survivors of 52 months. Finally, we evaluated this characteristic as a continuous variable rather than a predefined cutoff, as was done by most previous investigators.

Comparing the efficacy of primary treatments was not the aim of the present study and was not specifically tested. However, we can observe in our analyses that patients who experienced BCR after RT had a significantly higher CSM risk than those who experienced BCR after RP. Instead of reflecting treatment efficacy, these results likely reflect differences in the baseline characteristics between the groups. Moreover, we can speculate that these results suggest that the two definitions of BCR (PSA > 0.2 and PSA; PSA > 2 + nadir) are not comparable, and new definitions are warranted– perhaps based not only on a PSA cutoff but on PSA kinetics and other characteristics [30].

Our analyses were designed to evaluate the association of ttBCR with CSM within BCR patients and not to assess the impact of BCR itself; therefore, no conclusion on the latter can be drawn. Similarly, in our analyses, ADT was associated with higher CSM. However, as the study was not designed to assess the efficacy of ADT, this finding is most likely driven by confounding by indication, and no conclusions regarding the causal effect of ADT on mortality can be made.

From a clinical standpoint, our results suggest that the ttBCR should be included in future prediction tools for stratifying patients experiencing BCR after treatment with curative intent. Additionally, we contribute to the literature by creating conditional survival tables after BCR that can assist physicians in patient counseling and follow-up management for patients undergoing primary treatment for PCa. Indeed, current guidelines do not specify a recommended follow-up duration after primary treatment [2–5]. In this context, while further studies are warranted, our results suggest that oncological follow-up after both RT and RP could be safely discontinued after an adequate period for some patients (e.g. low- or intermediate-risk group) after a risk–benefit discussion with the patient. For instance, the 10-year predicted cancer-specific survival probability for a 65-year-old patient with an intermediate-risk PCa who experiences BCR after 10 years is > 0.95, which is significantly higher than the overall survival probability of the general population according to age (~ 0.83). This suggests that, in some patients, the oncological follow-up could be interrupted considering the limited life expectancy of these patients due to their age.

It is crucial to acknowledge the limitations of our study. As a retrospective cohort study, inherent biases and confounding factors may have been introduced. The study period was extensive, spanning several years during which significant modifications occurred in the diagnostic and treatment pathways for patients with PCa, including the introduction of multiparametric MRI and targeted biopsies for PCa diagnosis, as well as updates to the Gleason scoring system. All patients included in this cohort were Swedish. This may limit the generalizability of the findings, as ethnicity, healthcare delivery, and treatment practice patterns may differ across countries.

## Conclusion

Our findings demonstrate a strong association between ttBCR and CSM. These results suggest that ttBCR should be used to stratify PCa patients experiencing BCR after either RP or RT. The high survival probability for low- and intermediate-risk PCa who experience BCR after a long period following treatment should be considered for defining the intensity and duration of the oncological follow-up of these patients.

## Supplementary Information

Below is the link to the electronic supplementary material.

Supplementary file1 (DOCX 376 KB)

## Data Availability

The datasets generated during and/or analyzed during the current study are available from the corresponding author on reasonable request.
