# Supplementary material for: Time to BCR and cancer-specific mortality after radiotherapy or prostatectomy: implications for risk stratification and follow-up
Source: World J Urol. 2026 Feb 5;44(1):149. doi: 10.1007/s00345-026-06231-5 (PMC12876463; doi:10.1007/s00345-026-06231-5)
Supplement: Supplementary file 1 — Supplementary file1 (DOCX 376 KB) [file 345_2026_6231_MOESM1_ESM.docx]

**Supplementary material**

- **sTable 1.** Multivariable Cox models to assess the association between time to biochemical recurrence (BCR) and cancer-specific mortality in patients who experienced BCR after primary treatment.
- **sTable 2.** Characteristics of patients who underwent radical prostatectomy with complete information and experienced biochemical recurrence (BCR)
- **sTable 3.** Multivariable Cox model to assess the association between time to biochemical recurrence (BCR) and cancer-specific mortality in patients who experienced BCR after radical prostatectomy using pathological characteristics after excluding patients with incomplete pathological information (n= 1798).
- **sTable 4.** Multivariable Cox model to assess the association between time to biochemical recurrence (BCR) and cancer-specific mortality in patients who experienced BCR after radiotherapy plus adjuvant androgen deprivation therapy (n= 969).
- **sFigure 1**. Kaplan-Meier curves depicting cancer-specific survival probability according to primary treatment (A: radical prostatectomy; B: radiotherapy) and EAU risk group (low risk: green; intermediate risk: blue; high risk: red)
- **sFigure 2**. Predicted 10-year cancer-specific mortality by time to BCR (black lines) compared to 10-year any-cause mortality risk by age group (green: 55-59 years, yellow: 60-64 years, gray: 65-69 years, red: 70-74 years, blue: 75-79 years, purple: 80-85 years). Patients are grouped according to primary treatment and EAU risk group
- **sFigure 3.** Predicted cancer-specific survival probability for patients who experienced biochemical recurrence (BCR) after radical prostatectomy by time to BCR. Patients are grouped according to CAPRA-S risk group. The multivariable COX model used to calculate survival probabilities accounted for the non-linear relationship between time to BCR and cancer-specific mortality and was built with time to BCR, PSA at BCR, CAPRA-S risk group, year of primary treatment, adjuvant androgen deprivation therapy, and age at BCR

**sTable 1.** Multivariable Cox models to assess the association between time to biochemical recurrence (BCR) and cancer-specific mortality in patients who experienced BCR after primary treatment.

| **Characteristic** | **Radical Prostatectomy** | | | **Radiotherapy** | | |
| --- | --- | --- | --- | --- | --- | --- |
|  | **HR***^1^* | **95% CI***^1^* | **p-value** | **HR***^1^* | **95% CI***^1^* | **p-value** |
| **Time to BCR, per 1-yr increment** | **0.87** | **0.78, 0.97** | **0.012** | **0.69** | **0.64, 0.74** | **<0.001** |
| **PSA at BCR, per 5 ng/ml increment** | **1.04** | **1.03, 1.04** | **<0.001** | **1.01** | **1.01, 1.01** | **<0.001** |
| Clinical T stage |  |  |  |  |  |  |
| 1 | — | — |  | — | — |  |
| 2 | 1.53 | 1.01, 2.32 | 0.044 | 1.18 | 0.88, 1.58 | 0.3 |
| 3 | 0.92 | 0.44, 1.91 | 0.8 | 1.32 | 0.97, 1.80 | 0.077 |
| Clinical ISUP Grade Group |  |  |  |  |  |  |
| 1 | — | — |  | — | — |  |
| 2 | 1.86 | 0.99, 3.48 | 0.054 | 1.37 | 0.95, 1.97 | 0.095 |
| 3 | 4.23 | 2.30, 7.78 | <0.001 | 1.09 | 0.74, 1.61 | 0.7 |
| ≥4 | 4.61 | 2.50, 8.51 | <0.001 | 1.84 | 1.30, 2.61 | <0.001 |
| PSA at Diagnosis, ng/ml | 1.00 | 1.00, 1.01 | 0.2 | 1.00 | 1.00, 1.00 | 0.7 |
| Year of Treatment | 0.84 | 0.79, 0.90 | <0.001 | 0.92 | 0.88, 0.95 | <0.001 |
| Adjuvant Androgen Deprivation Therapy | 3.82 | 2.14, 6.84 | <0.001 | 2.26 | 1.52, 3.36 | <0.001 |
| Age at BCR, years | 1.02 | 0.98, 1.05 | 0.3 | 1.02 | 1.00, 1.04 | 0.019 |
| *^1^*HR = Hazard Ratio, CI = Confidence Interval | | | | | | |

**sTable 2**. Characteristics of patients who underwent radical prostatectomy with complete information and experienced biochemical recurrence (BCR)

| Characteristic | N = 1,798*^1^* |
| --- | --- |
| Age at BCR, years | 68 (63, 72) |
| PSA at Diagnosis, ng/ml | 8 (5, 12) |
| Pathological ISUP Grade Group |  |
| 1 | 198 (11%) |
| 2 | 575 (32%) |
| 3 | 600 (33%) |
| 4 | 211 (12%) |
| 5 | 214 (12%) |
| Pathological T stage |  |
| pT0 | 3 (0.2%) |
| pT2 | 747 (42%) |
| pT3 | 1,035 (58%) |
| pT4 | 13 (0.7%) |
| Positive Surgical Margins | 392 (22%) |
| Pathological T Stage |  |
| pN0 | 484 (27%) |
| pN1 | 126 (7.0%) |
| pNX | 1,188 (66%) |
| CAPRA-S Risk Category |  |
| Low Risk | 456 (25%) |
| Intermediate Risk | 945 (53%) |
| High Risk | 397 (22%) |
| Year of Treatment  (Median, range) | 2,012.0 (2,003.0, 2,020.0) |
| Adjuvant Androgen Deprivation Therapy | 96 (5.3%) |
| Time to BCR, months | 30 (12, 58) |
| PSA at BCR | 0.29 (0.23, 0.45) |
| *^1^*Median (IQR); n (%); Median (Range) | |

**sTable 3.** Multivariable Cox model to assess the association between time to biochemical recurrence (BCR) and cancer-specific mortality in patients who experienced BCR after radical prostatectomy using pathological characteristics after excluding patients with incomplete pathological information (n= 1798).

| **Characteristic** | **HR***^1^* | **95% CI***^1^* | **p-value** |
| --- | --- | --- | --- |
| **Time to BCR, per 1-yr increment** | **0.68** | **0.58, 0.80** | **<0.001** |
| **PSA at BCR, per 5 ng/ml increment** | **1.03** | **1.02, 1.04** | **<0.001** |
| Pathological ISUP Grade Group ≥3 | 1.10 | 0.66, 1.83 | 0.7 |
| Pathological T ≥3 | 2.50 | 1.30, 4.80 | 0.006 |
| Pathological N1 | 1.51 | 0.76, 2.99 | 0.2 |
| Positive Surgical Margins | 1.15 | 0.65, 2.02 | 0.6 |
| Year of Treatment | 0.86 | 0.80, 0.92 | <0.001 |
| Adjuvant Androgen Deprivation Therapy | 2.91 | 1.60, 5.29 | <0.001 |
| *^1^*HR = Hazard Ratio, CI = Confidence Interval | | | |

**sTable 4.** Multivariable Cox model to assess the association between time to biochemical recurrence (BCR) and cancer-specific mortality in patients who experienced BCR after radiotherapy plus adjuvant androgen deprivation therapy (n= 969).

| **Characteristic** | **HR***^1^* | **95% CI***^1^* | **p-value** |
| --- | --- | --- | --- |
| **Time to BCR, per 1-yr increment** | **0.69** | **0.64, 0.74** | **<0.001** |
| **PSA at BCR, 0.1 ng/ml increment** | **1.02** | **1.01, 1.02** | **<0.001** |
| Clinical T stage |  |  |  |
| 1 | — | — |  |
| 2 | 1.30 | 0.97, 1.75 | 0.077 |
| 3 | 1.43 | 1.05, 1.94 | 0.024 |
| Clinical ISUP Grade Group |  |  |  |
| 1 | — | — |  |
| 2 | 1.39 | 0.97, 2.01 | 0.075 |
| 3 | 1.20 | 0.81, 1.76 | 0.4 |
| ≥4 | 2.08 | 1.47, 2.94 | <0.001 |
| PSA at Diagnosis, ng/ml | 1.00 | 1.00, 1.00 | 0.6 |
| Year of Treatment | 0.92 | 0.89, 0.96 | <0.001 |
| Age at BCR, years | 1.02 | 1.00, 1.03 | 0.032 |
| *^1^*HR = Hazard Ratio, CI = Confidence Interval | | | |

**sFigure 1**. Kaplan-Meier curves depicting cancer-specific survival probability according to primary treatment (A: radical prostatectomy; B: radiotherapy) and EAU risk group (low risk: green; intermediate risk: blue; high risk: red)

**sFigure 2**. Predicted 10-year cancer-specific mortality by time to BCR (black lines) compared to 10-year any-cause mortality risk by age group (green: 55-59 years, yellow: 60-64 years, gray: 65-69 years, red: 70-74 years, blue: 75-79 years, purple: 80-85 years). Patients are grouped according to primary treatment and EAU risk group.

**sFigure 3.** Predicted cancer-specific survival probability for patients who experienced biochemical recurrence (BCR) after radical prostatectomy by time to BCR. Patients are grouped according to CAPRA-S risk group. The multivariable COX model used to calculate survival probabilities accounted for the non-linear relationship between time to BCR and cancer-specific mortality and was built with time to BCR, PSA at BCR, CAPRA-S risk group, year of primary treatment, adjuvant androgen deprivation therapy, and age at BCR
